# Offense and defense between streamers and customers in live commerce marketing: Protection motivation and information overload

**DOI:** 10.1371/journal.pone.0305585

**Published:** 2024-09-06

**Authors:** Junwei Cao, Lingling Zhong, Dong Liu, Guihua Zhang, Meng Shang

**Affiliations:** 1 School of Business, Yangzhou University, Yangzhou, China; 2 School of Business, Xinyang Normal University, Xinyang, China; 3 School of Flight, Anyang Institute of Technology, Anyang, China; SGH Warsaw School of Economics: Szkola Glowna Handlowa w Warszawie, POLAND

## Abstract

While live commerce provides consumers with a new shopping experience, it also leads them to experience shopping failures and to develop a self-protection mechanism to prevent wrong purchases. To address this issue, merchants have attempted to explore new marketing methods for live commerce, giving rise to an offense and defense game between streamers and consumers. In this study, we sought to confirm the effectiveness of consumer protection mechanisms and the impact of streamers’ information overload marketing strategy in live commerce. Accordingly, we constructed a hypothetical model based on protection motive theory and information overload theory. In addition, we analyzed the data from the simulated live streaming marketing on seven hundred people through partial least squares structural equation modeling. The results indicate that product utilitarian value uncertainty, consumers’ experiential efficacy, and response costs, which are the main factors in the formation of consumer protection mechanisms, influence consumers’ intention to stop their purchases. Streamers can circumvent consumer self-protection mechanisms through information overload marketing by reducing utilitarian value uncertainty and consumers’ experiential efficacy and increasing consumers’ response costs. However, consumers would be able to rebuild their self-protection mechanism through consumer resilience, which moderates the effects of information overload. This study’s results provide important theoretical perspectives and new ideas for formulating marketing strategies for live commerce.

## 1. Introduction

As digital transformation continues to evolve, digital platforms are becoming increasingly important for corporate performance growth [1]. Alongside the advancements in information technology, digital business models are continuously being updated. Especially with the development of information technology, e-commerce has gradually shifted to social commerce, the most representative form of which today is live commerce [2]. Live commerce not only facilitates product demonstrations and guided shopping but also creates an unprecedented platform that supports real-time communication about goods between streamers and consumers [3]. In live commerce, streamers interact with the audience in real-time and establish a relationship that stimulates consumers’ desire to participate. This interaction creates a better shopping experience for participants, making them more willing to purchase than they might be with traditional marketing methods [4, 5]. Consequently, consumers are increasingly favoring live streaming [6, 7].

China’s live streaming market is growing faster than that in any other region of the world. The size of China’s live commerce market exceeded $128.5 billion in 2020 [6]. According to a 2020 report released by Baidu.com, the frequency of searches for the keyword ’live streaming’ increased by 120% in one year. Some famous streamers have demonstrated significant business value; for example, a live shopping session can attract tens of millions of users and generate more than ten million dollars in revenue [8]. However, with the rapid growth of China’s live-streaming market, some problems have emerged.

In live commerce, consumers typically make consumption decisions through self-evaluation after receiving streamers’ recommendations and product descriptions [9]. However, there is an increasing number of cases where consumers exhibit impulsive buying behaviors, influenced by streamers to make unplanned purchases [10]. Many of these purchase behaviors are impulsive and conforming, leading consumers to acquire items they do not need or whose actual value is lower than expected [2, 11]. Over time, consumers develop self-protection mechanisms; that is, during subsequent live streaming sessions, they may hesitate or refrain from making purchases to avoid regrettable decisions [6, 12]. However, the psychological processes underlying these self-protective mechanisms are not fully understood. Hence, this study seeks to address the following question:

RQ1: What factors contribute to the emergence of consumer self-protection mechanisms in live commerce, and how do these mechanisms function to prevent incorrect purchasing decisions?

Notwithstanding such self-protection mechanisms, streamers can still influence consumer behavior through sophisticated communication strategies. For instance, streamers engage consumers with frequent interactions to retain them in the live broadcast room, subsequently providing comprehensive product information from various angles such as efficacy, design, price, usage, and suitability, ensuring consumers perceive a match between their needs and the product [7]. When consumers exhibit hesitation, streamers may create a false sense of urgency, emphasizing the product’s benefits and price advantages, promising additional free products, and using countdown timers to pressure consumers into making immediate purchases [5]. Moreover, streamers leverage parasocial relationships, persuading consumers by establishing a persona that garners recognition and sympathy, thereby fostering a sense of social connection [13]. The streamer is also good at creating a persona to gain consumer recognition and sympathy, which allows consumers to purchase in order to make social connections. They also ensure that the consumer treats them not as a provider of goods, but as a close friend. In addition, they can win consumers’ trust by influencing their emotional attachment or building intimacy [4, 12, 14, 15]. As a result, consumers may decide to follow the streamer’s shopping advice. They buy goods recommended by the streamer to maintain the relationship, even if they do not need the goods [5, 16]. Overall, streamers provide consumers with a lot of information in a short period by using a professional customer communication script to overcome the consumer’s protection mechanism against wrong purchases, reduce consumer hesitation, and lead them to make purchases. This marketing method based on "pushing” products in a "short time” and with a "large amount of information" meets the definition of information overload [17].

Merchants can influence consumer decision-making by affecting users’ psychological ownership. Information overload may serve as one method of control [18]. Numerous studies have explored the role of information overload in online marketing, focusing particularly on its relationship with consumers’ purchase intentions. However, the findings are mixed [19]. Some researchers argue that reducing consumer price sensitivity and increasing trust can encourage purchases [20], while others suggest that information overload heightens perceived risk among online consumers, reducing their intention to buy [19, 21]. Additionally, the impact of information overload on purchase intention may exhibit an inverted-U shape, varying with the level of overload [22]. Typically, the adverse subjective state induced by information overload can lead to suboptimal purchasing decisions [23]. Although the exact mechanism remains unclear, it could depend on consumers’ psychological evaluation processes in various shopping contexts. A recent study suggests that information overload indirectly influences purchasing behavior by affecting these psychological evaluations, such as inducing panic buying [24]. When confronted with information overload, consumers encounter more data than they can process, which heightens uncertainty and hampers decision-making. Some research suggests that postponing decisions, as a strategy to manage overload, fosters hopefulness. This newfound hope, resulting from extra time to deliberate and make informed choices, often leads to a preference for delayed but larger rewards, indicating a shift toward more patient and reflective decision-making [25]. Another study points out that virtual product displays in e-commerce introduce new challenges of information asymmetry, with the excess of digital information complicating consumer purchasing decisions [26]. For example, in online tourism promotion, the number of images in reviews, the frequency of merchant responses, and the length of these responses positively impact tourism product sales [27]. This leads to the inquiry: Does a similar mechanism operate in live commerce? How does information overload influence consumer self-protection against incorrect purchases in live commerce settings? Accordingly, the second question this study seeks to answer is as follows:

RQ2: In what ways does information overload marketing by streamers influence the effectiveness of consumer self-protection mechanisms in live commerce, and what are the specific tactics used by streamers to overcome these mechanisms?

In the context of information overload, individuals typically progress through three stages: compliance, acceptance, and resistance. Over time, consumers gradually mitigate the pressure of information overload and eventually re-establish their self-protection mechanisms to avoid erroneous purchases. To evaluate consumers’ capability to withstand information overload-induced marketing tactics, this study introduces the concept of resilience.

Resilience is acknowledged as an effective shield against stress. In the domain of information systems, prior research indicates that consumer resilience significantly contributes to alleviating stress [28]. This notion has also gained attention in consumer behavior research, suggesting its relevance in various contexts [29]. In live commerce, while merchants might breach consumers’ psychological defenses using information overload-centric marketing strategies, the potency of such strategies could be attenuated by consumer resilience. Prior investigations affirm that resilience can diminish the impacts of information overload [28]. However, in live commerce, when consumers are already under information overload, the role of resilience in restoring their self-protection mechanisms against wrong purchases remains unclear, which leads to the last research question of this study:

RQ3: How does consumer resilience interact with information overload in live commerce to affect the stability and effectiveness of consumer self-protection mechanisms, and what role does consumer resilience play in moderating the impact of streamer marketing tactics?

This study aims to investigate the dynamic interactions between streamers (sellers) and consumers within the live commerce environment, focusing on the streamers’ offensive strategies and the consumers’ defensive mechanisms. Offensively, streamers employ tactics designed to overcome consumer hesitation and resistance. These tactics include providing an excess of product information, creating a sense of urgency (e.g., limited-time offers), and emphasizing the benefits and features of the products. Such actions are considered offensive as they aim to break down the protective barriers consumers may have erected to prevent impulsive or misguided purchasing decisions. Defensively, consumers develop mechanisms to protect themselves from potential regrets associated with purchases. These include hesitating or abstaining from buying due to skepticism towards the streamer’s presentation and coping with the streamer’s aggressive sales tactics, often characterized by "information overload." The study explores how streamers successfully influence consumer decisions (offense) and how consumers, in turn, protect themselves from making decisions they might regret (defense). This dynamic interplay of "offense" and "defense" forms the core of this research. By analyzing these interactions, the study seeks to reveal how these mechanisms jointly affect consumer behavior on live commerce platforms and how they shape the unique consumer experience in live commerce. It aims to provide a comprehensive understanding of how consumer protection mechanisms are established and function and how they impact the streamer’s information delivery strategies.

As live commerce swiftly expands, consumers often find themselves overwhelmed by the vast amount of product information available. This study aims to explore the interaction between information overload and consumer resilience, and how these factors influence consumer purchasing decisions. By developing a theoretical model, this research not only clarifies how consumers establish protective mechanisms (defense) to counteract aggressive marketing strategies in a live streaming environment but also examines how streamers can influence these mechanisms through their information strategies (offense). This approach offers a new perspective on consumer behavior in the digital marketplace.

The innovative aspects of this study are manifold. 1) it provides empirical evidence of the existence of psychological self-protection mechanisms among consumers in live commerce, thus enriching our understanding of consumer behavior within the context of social commerce. These insights reveal how consumers protect themselves from making erroneous purchases and navigate the rapidly changing shopping environment. 2) the study enhances our comprehension of the role of information overload in influencing consumer behavior, contributing valuable insights to the marketing literature. It offers strategic recommendations for e-commerce platforms on how to balance the provision of information with consumers’ ability to process it, aiming to enhance consumer purchase intentions and satisfaction. 3) By identifying consumer resilience as a moderating factor in the dynamic interaction between streamers and consumers, this research extends existing knowledge within consumer behavior studies. It highlights the importance of resilience in helping consumers withstand persuasive marketing tactics, providing practical insights for developing more effective marketing strategies that tackle the challenges of information overload in digital environments. Overall, this study not only aids in crafting more humane marketing strategies but also promotes the protection of consumer rights, contributing to the sustainable development of the e-commerce industry.

## 2. Theoretical background and hypothesis development

### 2.1 Consumer’s defense mechanisms: Protection motivation theory

Protection motivation theory (PMT) was proposed by Rogers [30]. It divides the process by which people become motivated to protect themselves into three stages: threat appraisal, coping appraisal, and protective behavior. Individuals often develop risk-averse protective intentions to protect themselves from harm (e.g., natural disasters, the threat of global climate change). However, before taking any final protective actions, they usually weigh the benefits and risks of the action by comparing the level of environmental threat and their ability to cope [30].

Threat appraisal is a cognitive process through which individuals measure the threat level. It includes two aspects: perceived severity and perceived susceptibility [31]. The perceived severity of threat refers to an individual’s judgment of the severity of harm. Perceived susceptibility reflects an individual’s perception of the possibility of harm. Individuals’ perceptions of threat severity and susceptibility can motivate protective behavior [30].

Coping appraisal refers to the assessment of an individual’s ability to exhibit risk-prevention behaviors. It includes three aspects: self-efficacy, response efficacy, and response cost [30]. Self-efficacy is the individual’s judgment of their ability to display the desired behavior [32]. Response efficacy is the expectation regarding the outcome of an individual’s protective action [30]. Response costs are the benefits lost by individuals who engage in risk-prevention behaviors [30]. The sum of an individual’s self-efficacy and response efficacy minus the required response cost constitutes the results of the coping appraisal. The higher the response efficacy and self-efficacy and the lower the response cost, the more likely it is that the individual will decide to engage in protective behaviors [33].

PMT was mainly used for research in healthcare areas such as vaccination [34, 35] and disease management [36]. It was later extended to the study of management information systems, such as network security behavior [37] and information security behavior [38, 39]. It has also recently been used in studies of consumer purchasing behavior. For example, an investigation suggested that COVID-19 influences customers’ willingness to buy clothes by affecting perceived severity and self-efficacy in relation to the disease [40]. Another study suggested that the perceived severity of environmental problems and the effectiveness of environment protection responses motivate consumers to engage in protection behaviors and ultimately influence their purchasing behavior in relation to green products [41]. Another PMT-based study indicated that perceived risk positively influences consumers’ motivation to spend money in luxury restaurants [42]. In summary, these previous studies confirm that risk factors from the living environment affect consumers’ consumption attitudes. Risk factors induce product uncertainty, and consumers are more likely to be uncertain about the value of products in such cases. For this reason, consumers may decide to protect themselves by considering the risks and benefits of their purchases [42], which may cause them to stop buying or to indulge in panic buying [43].

From the PMT perspective, in live streaming, the consumer protection mechanism against wrong purchases consists of three components: an assessment of the severity of the threat factors that may lead to wrong purchases; an assessment of the possibility of a response; and ultimately, the act of interrupting the purchase. With a wide variety of products appearing on live streaming, it is understandable for consumers to be uncertain whether the purchased products will be worthwhile. Therefore, they have to evaluate the risk of uncertainty, analyze the advantages and disadvantages to buying or not buying the product, and protect themselves from making incorrect purchases.

#### 2.1.1 Threat appraisal

The essence of live commerce is the sale of goods. Live commerce can display the utilitarian value of goods in a superior way, compared to traditional e-commerce. Streamers can reduce the uncertainty regarding product value based on social and product attributes. They improve customers’ purchase intentions through real-time interaction and the display of product information [3, 6]. The streamer can use various methods to provide consumers with detailed and vivid product information. Consumers obtain product information that seems immediate, easily comprehensible, and inspirational. They immerse themselves in a pleasant online shopping atmosphere and eventually engage in purchase behavior [2, 7, 13]. In addition, streamers can quickly establish social relationships with customers based on mutual benefits by communicating with consumers from their perspective and responding to consumers’ needs instantly [13]. This social relationship increases consumers’ trust in the streamer, supports the hedonic value consumers derive from live streaming, creates emotional commitment and attachment to the streamer in consumers, and promotes their willingness to purchase [4, 12].

Since live commerce demonstrates the value of products to consumers efficiently and reduces the uncertainty in consumers’ perception of the value of products, consumers’ willingness to purchase improves [3, 6]. For example, when a consumer feels that the product does not justify the value and that the purchase of the product does not necessarily increase the intimacy with the streamer, they reduce their purchase expectations and stop the purchase. From the PMT perspective, the higher the uncertainty in consumers about the value of a product, the more they realize that the current shopping environment is causing them to make wrong purchases, and the higher the possibility that they will suffer losses if they continue to buy.

Product value uncertainty is defined as the degree of difficulty consumers have in assessing product attributes and predicting future product performance [44]. Product uncertainty is a major barrier in online shopping. It has a significant impact on consumers’ willingness to buy [45]. In a live commerce environment, consumers tend to be unsure of whether the products are worth purchasing, and therefore, they may evaluate the risks in continuing to purchase [42].

Product value uncertainty, a multifaceted construct, encompasses uncertainties related to product description, fit, and performance. Specifically, product description uncertainty arises when the seller’s presentation fails to adequately encapsulate the product’s characteristics. Product performance uncertainty is the consumer’s apprehension about the actual performance aligning with their expectations [44], while fit uncertainty pertains to the consumer’s concern about the product meeting their specific needs [46]. In the realm of live commerce, where the immediacy and interactivity of the shopping experience are pronounced, the clarity of product value, particularly its utilitarian aspect, is paramount. Utilitarian value, the assessment of a product’s functional benefits [47], significantly influences consumer purchase behavior [48] and is even more critical in the dynamic environment of live commerce [49]. The unique capabilities of live commerce to demonstrate products and deliver information efficiently are pivotal in eliciting consumers’ utilitarian shopping motivation [50].

However, when faced with product value uncertainty in this live interactive setting, consumers engage in a threat appraisal process, as postulated by Protection Motivation Theory (PMT). This process involves evaluating the severity of the potential threat (e.g., the risk of dissatisfaction or regret from an incorrect purchase) and their susceptibility to this threat (e.g., the likelihood of making a poor purchase decision due to inadequate product information). Consequently, this perceived threat may catalyze a protective behavioral response, wherein consumers are inclined to halt their purchasing decision to safeguard against potential adverse outcomes. Therefore, integrating the constructs of PMT with the dynamics of live commerce, we propose the following hypothesis:

H1a: In live commerce, uncertainty about a product’s utilitarian value positively affects consumers’ intention to stop purchase.

Live commerce, distinct from traditional e-commerce, thrives on the interactive dynamics between streamers and consumers, often resembling a celebrity-fan relationship [50]. This interactive milieu fosters a hedonic value proposition, where the enjoyment and experiential benefits derived from the engagement are paramount [51].

The hedonic value in live commerce is not merely about the product but also about the relational experience with the streamer, enhancing trust, emotional commitment, and a sense of community among consumers (Hu & Chaudhry, 2020; Park & Lin, 2020). Previous research has confirmed that hedonic value is an important motivation in consumers’ purchase decisions during live commerce [50, 52]. Live commerce transactions with a hedonic value can influence customer attitudes and behavioral responses. For example, when consumers perceive the integrity and kindness of a streamer through live streaming, they begin to trust the streamer. They believe that they can help streamers by purchasing products [53].

In the framework of Protection Motivation Theory (PMT), the concept of hedonic value uncertainty in live commerce can be interpreted as a form of threat appraisal. When consumers are unsure about the hedonic benefits of their engagement in live commerce—whether due to ambiguous streamer-consumer interactions, inconsistent content quality, or unclear emotional rewards—they undergo a cognitive process of assessing the severity of this uncertainty and their vulnerability to potential dissatisfaction or regret associated with their purchase decisions.

Given this backdrop, hedonic value uncertainty can trigger protective motivations, prompting consumers to reconsider or halt their purchase intentions as a mechanism to shield themselves from the anticipated dissonance of unmet emotional expectations or the lack of perceived relational value from the live commerce experience. Therefore, synthesizing the aspects of PMT and the hedonic nuances of live commerce, we propose the following hypothesis:

H1b: In live commerce, hedonic value uncertainty positively impacts consumers’ intention to stop purchase.

#### 2.1.2 Coping appraisal

In the context of live commerce, consumers’ decision-making processes are profoundly influenced by their past purchasing experiences, which shape their perceptions of self-efficacy and response efficacy—key components of the coping appraisal mechanism as delineated in PMT. Self-efficacy in this realm refers to consumers’ belief in their ability to make informed purchasing decisions, while response efficacy pertains to their assessment of the effectiveness of these decisions in yielding satisfactory outcomes [54]. Experiential efficacy, a construct synthesizing self-efficacy and response efficacy, encapsulates consumers’ confidence and perceived effectiveness based on their historical interactions within the live commerce environment. This concept aligns with PMT’s coping appraisal, where individuals evaluate their capability to mitigate or avoid perceived threats (in this case, the threat of unsatisfactory purchases).

Empirical studies underscore the link between past purchasing experiences and future purchasing behaviors, Studies suggesting that positive experiences enhance consumers’ confidence and perceived control over future transactions, potentially reducing their likelihood to halt purchases due to uncertainty [55, 56]. Based on previous research, we combined self-efficacy and response efficacy as experiential efficacy in this study. Therefore, we propose the following hypothesis:

H2a: In live commerce, consumers’ experiential efficacy has a positive impact on their intention to stop purchase.

Protection Motivation Theory (PMT) provides a robust framework for understanding how individuals assess and respond to threats, incorporating elements of threat appraisal and coping appraisal. In the context of live commerce, consumers engage in a threat appraisal process, where they evaluate the potential risks associated with their purchasing decisions. Concurrently, the coping appraisal process involves an assessment of the response costs associated with taking protective actions to mitigate these risks. According to PMT, the likelihood of an individual engaging in a protective behavior increases when the perceived severity and vulnerability associated with the threat are high, and when the perceived response efficacy and self-efficacy are significant enough to outweigh the response costs.

In live commerce settings, streamers create value bonds with consumers by offering extra benefits such as personalized recommendations, product trials, and exclusive offers, as highlighted by [13]. These value bonds can be viewed as factors that lower the perceived response costs of continuing a purchase, as they enhance the perceived benefits of engaging with the live commerce platform and reduce the perceived gains of discontinuing a purchase. When consumers contemplate stopping a purchase, they weigh the response costs, which now include potential losses of additional benefits and the emotional connection with the streamer. If these perceived response costs of protective action (i.e., stopping the purchase) are viewed as high relative to the benefits of risky behavior (continuing the purchase despite potential risks), the consumer’s motivation to adopt self-protective behavior is likely to diminish. Therefore, we propose the following hypothesis that directly links the PMT constructs with the context of live commerce:

H2b: In live commerce, consumers’ perceived response costs have a negative impact on their intention to stop buying.

### 2.2 Streamer offense strategy: Information overload

#### 2.2.1 Information overload and marketing

In recent years, terms such as information asymmetry, data smog, and information overload have frequently appeared in research reports. However, information overload has long been an important issue in daily life. Information overload means that the amount of information processing exceeds the capacity for information processing [57]. In the process of information retrieval, analysis, and decision making, information overload occurs when the amount of information that needs to be processed is greater than an individual’s ability to process information in a short period of time [58]. The degree of individual information overload depends not only on the amount of information received but also on the nature of the information and the individual’s experience reserve. When an individual’s experience is insufficient to cope with uncertain and complex information, the information overload phenomenon is more significant [59]. An information overload is harmful to individuals. It confuses them, affects their ability to prioritize, and makes it difficult to use previous information effectively. As a result, it leads to poor decision-making, dysfunction, and anxiety [59, 60]. In particular, information overload from social media is effective in changing consumer attitudes and convincing them to believe the message maker’s opinion [61].

Early research on information overload in traditional retail marketing suggests that the relationship between the quantity of information and the quality of purchase decisions has an inverted U-shaped relationship [62]. However, subsequent studies have pointed out that an increase in the amount of information does not influence consumer decision-making and actual purchase behavior [63, 64]. However, an increase in the quantity of information can reduce the accuracy of purchase decisions [65].

With the development of e-commerce, the impact of information overload in the online environment on consumers’ purchase intentions has been studied. Chen, Shang and Kao [23] suggested that e-retailers can deliver rich information to customers, but information overload caused by too much information could cause consumers to slip into a poor subjective state when making decisions. A study suggested that more product information, whether about the product or its price, would increase consumer trust, reduce consumer price awareness, and lead to consumer purchases [20]. Interestingly, a survey of 1,396 online shoppers in Spain confirmed that information overload positively influences consumers’ willingness to buy online, but also increases perceived risk, and indirectly decreases their willingness to buy [19]. According to the study’s authors, their findings "add some controversy to the relationship between information overload and customer purchase intentions" [19]. Subsequently, [22] conducted a study on the purchase intention toward online experience services and suggested an inverted U-shaped relationship between information load, trust, and purchase intention. That is, low information load is ineffective in fostering trust and purchase intention; medium information load is effective in fostering trust and purchase intention; and high information load is less effective than medium information load in fostering trust and purchase intention. However, a subsequent study showed that information overload reduces consumer trust and purchase intention, especially in online mobile shopping [21]. In summary, there are conflicting views on the role of information overload marketing in the field of e-commerce, and further research is needed.

#### 2.2.2 Information overload and live commerce

Product uncertainty is a major barrier to online shopping, and product descriptions and third-party product warranties help reduce product uncertainty [44]. Live commerce allows streamers to present detailed and vivid product information to consumers as if they were presenting this information in the presence of the consumer [2, 7, 13]. This ultimately reduces the uncertainty about the utilitarian value of the product.

Streamers also communicate with consumers from the consumer’s perspective and establish a social relationship of mutual understanding [13], thereby delivering the hedonic value of live streaming to consumers. Moreover, consumers gradually form emotional commitments and attachments to streamers [4, 12], following which their hedonic value uncertainty decreases, and they become willing to purchase goods to enhance their relationship with the streamer.

When streamers find that consumers are hesitant to buy, they urge them to buy, repeatedly emphasizing the effectiveness of the product and the price advantage (utilitarian information), so that consumers are afraid of missing the opportunity to buy. Streamers deliberately limit the number of discounted products, replenish goods on the grounds of intimacy, and use rounds of surprises to increase consumers’ emotional attachment (hedonic information). In this time limitation created by streamers, the utilitarian and hedonic information greatly exceeds the consumer’s information processing capacity, and consumers enter a state of information overload.

Information Overload Theory posits that an excess of information can overwhelm consumers, impairing their decision-making capabilities [59]. In the context of live commerce, streamers can exacerbate information overload by rapidly presenting product information, thereby challenging consumers’ ability to process and evaluate this information effectively. This overload can trigger mechanisms like information avoidance and the information cocoon effect, where consumers limit their information sources to those that are most immediately accessible or reassuring—in this case, the streamer [66].

From the perspective of PMT, information overload can impact the threat and coping appraisal processes. When consumers face information overload, their ability to assess the potential threat (e.g., making a wrong purchase decision) and their efficacy in coping with this threat (e.g., evaluating product value accurately) can be compromised. Consequently, they may rely more heavily on the streamer’s guidance, which can reduce their perception of uncertainty regarding the product’s utilitarian and hedonic values. Thus, information overload in live commerce can paradoxically reduce consumers’ perceived uncertainty about a product’s value by nudging them towards a simplified decision-making process that leans heavily on the streamer’s input. This dynamic suggests that information overload might inadvertently diminish consumers’ perceived utilitarian and hedonic value uncertainty, as they become more dependent on the streamer’s narratives and less inclined to seek out additional information or evaluate their options critically. Therefore, the following hypothesis was proposed in this study:

H3a: Information overload has a negative effect on utilitarian value uncertainty.
H3b: Information overload has a negative effect on hedonic value uncertainty.

Protection Motivation Theory (PMT) provides a framework for understanding how individuals appraise threats and their coping responses. In the context of live commerce, experiential efficacy—consumers’ belief in their ability to make informed purchase decisions based on past experiences—is a crucial component of the coping appraisal process. Information Overload Theory suggests that an excess of information can impede individuals’ ability to process and make decisions, potentially undermining their experiential efficacy [59]. When consumers encounter information overload in live commerce, their ability to leverage past purchasing experiences may be compromised, leading to cognitive dissonance and a reduction in self-efficacy [67]. This phenomenon is exacerbated by streamers who rapidly disseminate product information, creating a sense of urgency and compelling consumers to make quick decisions, often sidelining their past experiences and judgment.

Additionally, response cost, a concept from PMT that denotes the perceived cost associated with engaging in a protective behavior, can be influenced by information overload. In live commerce settings, streamers accentuate discounts and limited-time offers, inflating the perceived cost of not making a purchase and thus potentially reducing consumers’ likelihood of engaging in protective behaviors like delaying or forgoing a purchase. Therefore, this study proposes the following hypotheses:

H4a: Information overload has a negative effect on experiential efficacy.
H4b: Information overload has a positive effect on response cost.

### 2.3 Consumer defense mechanism recovery: Consumer resilience

Resilience, defined as the ability of an individual to recover in the face of pressure or adversity, exhibits significant individual differences [68]. In the business realm, particularly in consumer behavior research, resilience has become a critical concept. In the business environment, marketing strategies may constrain consumers’ freedom, prompting them to face various temptations, with consumer resilience emerging as a key force to resist these market temptations and maintain individual freedom of choice [69]. Furthermore, consumer resilience displayed in shopping environments is often influenced by personal intrinsic traits and family environment, which together constitute important dimensions affecting consumer decisions [68].

In the emerging field of live commerce, consumer resilience manifests as particularly complex, influenced by multiple factors. Chen and Yang [70] study reveals the intrinsic connection between consumer experience and purchase intent, emphasizing the mediating role of network structural embeddedness in this process, pointing out that optimizing the usability of network interfaces and relationship services significantly impacts enhancing consumer resilience. Additionally, Xu, Cui and Lyu [26] explore the influence of host attributes and consumer interaction on purchasing behavior in live e-commerce, demonstrating how consumer trust and social capital play crucial roles in building consumer resilience. Zhang, Qi and Lyu [9] and Zhang, Yang and Bei [18] approach from the perspective of virtual communities, exploring the roles of knowledge sharing and social capital in promoting consumer resilience. Wei, Hai, Zhu and Lyu [25] emphasize the importance of perceived information integrity in shaping consumer resilience in studies of consumer delay behavior. Finally, Helin, Donglu, Shaoying, Decheng and Bei [27] analyze from the perspective of online reviews and merchant interaction responses how these factors influence consumer trust in brands and willingness to continue purchasing, providing new insights into understanding consumer resilience in live commerce. Together, these studies constitute an in-depth understanding of consumer resilience in live commerce, revealing its multiple influencing factors.

There may also be a relationship between information overload and consumer resilience. Information overload makes consumers dyscognitive dissonance, vulnerable, and susceptible; however, its impact can be reduced by consumer resilience [28]. When consumers experience information pressure, their original thought equilibrium may be disrupted. However, resilience can reduce information pressure. Consumers with high resilience feel less information pressure [28]. In particular, consumers with high resilience may recover more quickly from a potentially stressful event (e.g., service failure) compared to low resilience consumers [71].

In the field of consumer behavior research, the role of consumer resilience in reducing product uncertainty is unclear. However, a study in healthcare suggested that resilience in healthcare systems is beneficial in reducing risk uncertainty [72], who found resilience to be beneficial in reducing risk uncertainty in healthcare systems, we suggest that resilience in consumers can similarly reduce uncertainty in purchasing decisions by enhancing their ability to process overwhelming information and make clear evaluations of potential risks and benefits. Further, [73]. highlight the role of resilience in reducing the cost of adopting reliability behaviors. Extending this insight to the consumer domain, we argue that resilience enables consumers to better cope with information overload, thereby reducing the cognitive costs associated with evaluating information and making purchasing decisions. Consequently, resilient consumers are better equipped to discern the utilitarian and hedonic values of products and to assess the efficacy of their purchasing decisions, even in the face of excessive information. Therefore, we propose the following hypotheses:

H5a: Consumer resilience reduces the negative effect of information overload on utilitarian value uncertainty.
H5b: Consumer resilience reduces the negative effect of information overload on hedonic value uncertainty.
H5c: Consumer resilience reduces the negative effect of information overload on experiential efficacy.
H5d: Consumer resilience reduces the positive relationship of information overload on response costs.

## 3. Research model and investigation design

### 3.1 Research model

**Fig 1:**
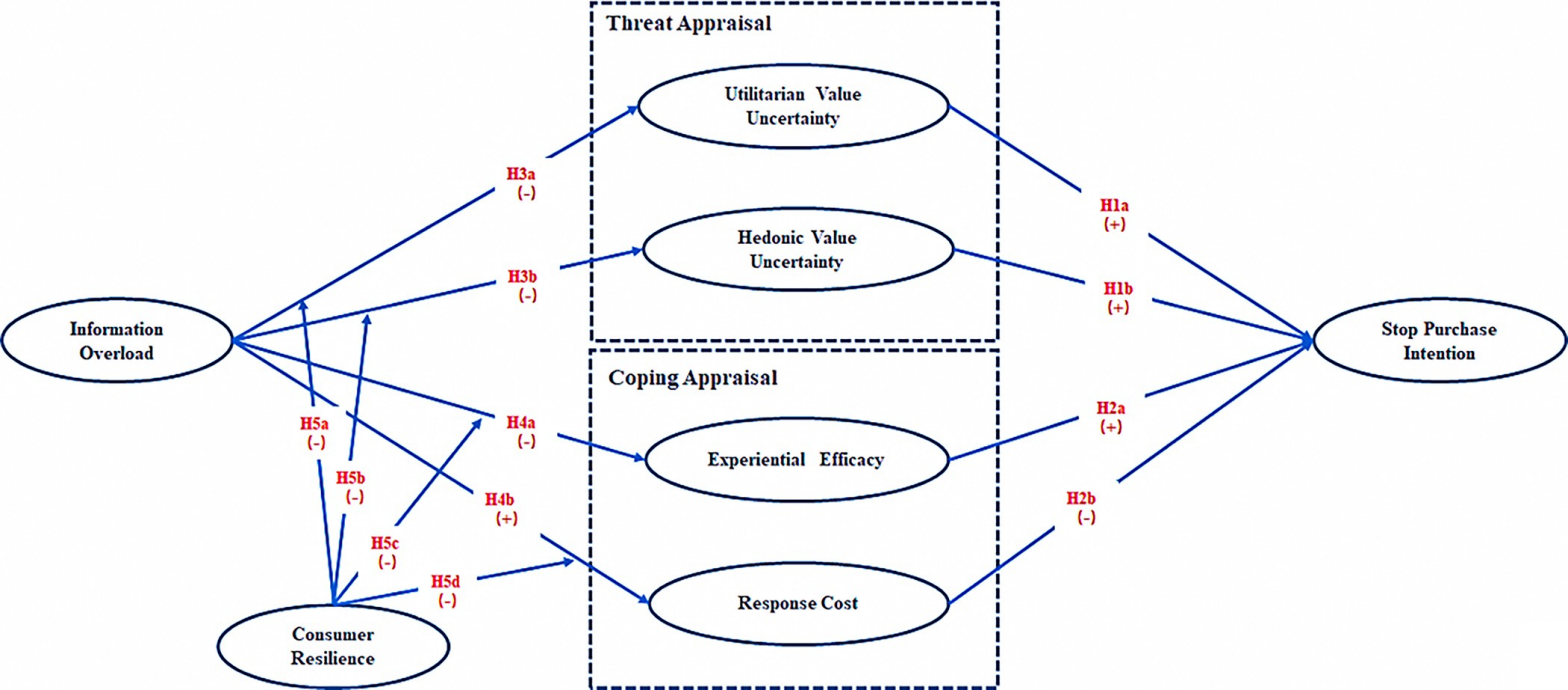
Research model.

Based on the theoretical foundations and hypotheses discussed thus far, this study proposes a research model as shown in Fig 1. First, we construct a consumer protection mechanism to prevent incorrect purchases in live commerce based on the PMT. The PMT proposes that consumers’ perceived threat severity and susceptibility can be used for threat appraisal. Self-efficacy, response efficacy, and response cost can be used for coping appraisal. In this study, in the context of live commerce, utilitarian and hedonic value uncertainty were used to assess the severity of and susceptibility to wrong purchases, and experiential efficacy and response cost were used to measure consumer coping appraisal. Together, these factors influence consumers’ self-protective behavior. However, the investigation of consumer behavior through self-reporting may bias the results. Conversely, the intention of consumers can easily be measured through self-reported data [67, 74]. Ajzen [75] indicated that the variables in PMT influence consumers’ behavior by affecting their intentions, and consumers’ intentions significantly predict their behavior. Accordingly, this study focused on consumers’ intentions to stop purchases rather than their actual behavior. Second, information overload, as an attacking tool used by streamers to break through consumers’ psychological protection, was introduced into this model. Its function in live commerce has been confirmed. Finally, consumer resilience was introduced into the model as a moderating variable, and its role in the consumer’s reconstruction of self-protection mechanisms was also confirmed.

### 3.2 Simulation environment design and survey

Due to the fact that retrospective surveys are detached from the actual situation, they can introduce some biases into the research. Therefore, to minimize errors, we designed and simulated a live streaming marketing environment to aid the survey., and the effectiveness of this method has been proved by previous studies of consumer psychology [26]. As consumers are not always in an information overload environment, asking them to respond to a questionnaire by recalling their most recent experience of information overload would result in measurement bias. The longer they are away from the information overload environment, the greater the bias. Additionally, in a pressure environment, it is necessary to accurately measure the effect of consumer resilience. Therefore, we simulated a live streaming environment to validate the model proposed in this study. In the full simulation environment, participants were completely anonymized, participants were informed of the purpose of the simulated live streaming marketing, only the necessary data were collected and kept strictly confidential, and certain rewards were given after the simulated live streaming marketing; informed consent was obtained by having a representative of the participant sign an informed consent form.

Simulation environment. (1) Streamer marketing encompasses three stages: interaction, product introduction, and transaction promotion. During the interaction and product introduction stages, the streamer must create an atmosphere to promote interaction and introduce the product. In the stage of promoting the transaction, the streamer must focus on utilitarian and hedonic information; they also use professional customer communication scripts by repeatedly emphasizing the effects of the product, thereby creating an atmosphere of a limited-time discount, urging consumers to immediately place orders, and as far as possible, exposing consumers to an information overload. According to these different stages, we compiled sample phrases for streamer reference (see supporting information S1 Appendix). (2) According to Baidu index data (http://index.baidu.com), people aged 20–39 years in Jiangsu province of China were most concerned about live commerce in December 2021. So, this part of the population was taken as a recruitment target for participation in the live streaming marketing simulation. (3) Two streamers (one male and one female) were recruited. They were well-versed with using professional customer communication scripts for live streaming and had six to nine months of live commerce experience; each person received a commission of 1000 RMB (about 150 USD). (4) Seven imperceptibly branded products that are suitable for both men and women were used in the simulated live streaming environment. (5) Two online meeting channels, A and B, were prepared to simulate a live room. (6) Questionnaire scales validated in previous studies were used in the present study, with the questions adapted to the present context. The participants chose responses on a 5-point Likert scale; the questionnaire was reviewed and revised by experts in the field, yielding the final questionnaire used as shown in S2 Appendix.

Simulating the process of live streaming. (1) The participants entered the online meeting channel A, and the two streamers worked with each other to interact with the participants on the channel, as the staff provided each participant with a reward of 10 RMB (about 1.5 USD). When the number of participants reached 100, we closed the entry to Channel A, and the simulated live streaming marketing began. (2) The two streamers began to work with each other to introduce the product for 5 minutes. During this time, if participants thought that the product may not be suitable for them and they intended to stop purchase, they were asked to inform the staff and were invited to wait in online meeting channel B. They were then told that they could participate in a prize-draw by completing the next phase of the trial. (3) When the activity of channel A was finished, the two streamers entered channel B and started marketing by "pushing the deal.” They had to do their best to allow consumers to experience information overload. About 3 minutes later, participants were listening to streamer and filling out the questionnaires we prepared at the same time. After completing the questionnaires, the participants were given a gift.

We conducted seven simulations of live streaming marketing in January 2022; the details of simulations of live streaming marketing are listed in Table 1. A total of 391 valid questionnaires were returned.

**Table 1:** Simulated live streaming marketing details.

Date	Products	Number of people (channel A)	Number of people (channel B)	Returned questionnaires	Valid questionnaires
Jan. 3	Assembly models	100	71	70	67
Jan. 4	Mobile hard drives	100	57	52	44
Jan. 5	Bluetooth speakers	100	63	63	59
Jan. 6	Game handhelds	100	73	72	70
Jan. 7	Running shoes	100	57	57	53
Jan. 8	Induction cookers	100	62	60	57
Jan. 9	Leisure backpacks	100	59	54	41
Total		700	442	428	391

## 4. Empirical analyses

### 4.1 Analysis methods

We derived the descriptive statistics of the sample and assessed the indicators related to data quality. The proposed hypotheses were tested using a research model.

There are two types of structural equation models: the covariance-based structural equation model (CB-SEM) and variance-based partial least squares structural equation model (PLS-SEM). In this study, PLS-SEM and the corresponding software package SmartPls 3.0 were used for data analysis [76]. The main reasons are: (1) Compared with CB-SEM, PLS-SEM is more suitable for measuring complex models, especially those with more than six variables [77]. There are seven variables in this study. (2) Compared to CB-SEM, PLS-SEM can calculate non-normal distribution data more effectively [77]. A multivariate normality analysis was performed on the data using a web-based calculator (http://www.biosoft.hacettepe.edu.tr/MVN/) [78]. The results showed Mardie’s multivariate skewness (β = 29.297, p <0.05) and multivariate kurtosis (β = 470.706, p <0.001), indicating that the data in this study were multivariate non-normal [79]. (3) PLS-SEM is more suitable for small-sample measurements [77]. In summary, PLS-SEM was more suitable for analysis in this study.

### 4.2 Measurement

Prior to developing scales to measure the variables, we operationally defined the variables. 1) Product Utilitarian Value Uncertainty: This variable refers to the consumer’s perceived difficulty in assessing the attributes and future performance of a product showcased in live commerce. It signifies the degree to which consumers feel uncertain about the practical and functional value of a product. 2) Hedonic Value Uncertainty: This variable measures the consumer’s perceived uncertainty regarding the enjoyment and experiential benefits they will receive from purchasing a product during a live commerce session. It reflects the ambiguity consumers feel about the emotional and experiential returns of their purchase. 3) Experiential Efficacy: This combines self-efficacy and response efficacy, representing the consumer’s belief in their ability to make correct purchase decisions based on past experiences in similar contexts. 4) Response Costs: This variable reflects the perceived losses or costs associated with halting a purchase during a live commerce session. It includes the potential loss of additional benefits or emotional connections with the streamer. 5) Information Overload: In the context of this study, information overload refers to a marketing strategy employed by streamers where a vast amount of product information is delivered rapidly, surpassing the consumer’s ability to process it effectively. This overload aims to influence purchase decisions by creating a sense of urgency and reducing the consumer’s ability to evaluate the product critically. 6) Consumer Resilience: This variable measures the consumer’s ability to withstand and recover from the stress induced by information overload in live commerce. It indicates the consumer’s capacity to maintain or regain their self-protection mechanisms against incorrect purchases despite the overwhelming information presented by streamers.

To enhance the methodological rigor and ensure the content validity of our research, the scales implemented in this study were meticulously derived from extant scholarly literature, with necessary terminological modifications to tailor them to our specific research context. Specifically, the constructs of utilitarian value uncertainty and hedonic value uncertainty were adapted from Lu and Chen [6] and Park and Lin [12]. Additionally, the concept of experience efficacy was redefined based on the frameworks proposed by Farooq, Laato, Islam and Isoaho [32] and Tsai, Jiang, Alhabash, LaRose, Rifon and Cotten [37], while the measure of response cost drew upon the operationalization by Farooq, Laato, Islam and Isoaho [32]. Furthermore, the construct of Purchase Interruption Intention was refined following the methodology of Park and Lin [12], with the notion of information overload being recalibrated based on Farooq, Laato, Islam and Isoaho [32]. The dimension of consumer resilience was reconceptualized in accordance with Bermes [28].

Acknowledging the geographical specificity of our data collection in China, we employed the back-translation method to ensure linguistic and conceptual equivalence. Initially, the first author translated the original English questionnaire into Chinese, which was then back-translated into English by an independent translator unfamiliar with the study’s objectives. This iterative process facilitated a meticulous comparison of the two English versions, confirming their consistency without significant discrepancies.

To further ascertain the face validity of the instrument, we engaged three doctoral candidates specializing in Marketing and two experts in marketing to scrutinize each item for potential ambiguities. Their insights contributed to the refinement of the instrument. Subsequently, a pilot study involving 61 participants with prior experience in live commerce was conducted to validate the scale’s reliability. The details of the refined scale and its validation are documented in S2 Appendix of the Supporting Information.

According to Baidu index data (http://index.baidu.com), people aged 20–39 years in Jiangsu province of China were most concerned about live commerce in December 2021. Therefore, to obtain a representative sample, this study employed a combined methodology of random sampling and snowball sampling for data collection. During the random sampling phase, the research team randomly selected live streaming rooms and SNS platforms, engaging with the audience during or after the live streaming session to inquire about their willingness to participate in this study. This process was designed to ensure the randomness of the sampling, allowing the sample to broadly reflect the characteristics of the target population. In the snowball sampling approach, participants who consented to partake in the study were encouraged to invite their friends to join the research. These friends were required to meet the same participation criteria: being between the ages of 20 and 29 and having experience with live commerce. Through this method, the research team was able to reach a broader audience, particularly targeting individuals who might not frequently appear in the randomly selected live streaming rooms. Ultimately, study pre-recruited 1326 people aged 20–39 from Jiangsu province who have previously experienced wrong purchases during live streaming.

### 4.3 Demographics and bias test

A total of 391 valid questionnaires were collected. Among the participants, 202 (51.7%) were male and 189 (48.3%) were female; 182 (46.5%) were 20–29 years old and 209 (53.5%) were 30–39 years old; 132 (33.8%) had a college degree and 103 (26.3%) had a bachelor’s degree; and the largest number of people earned RMB 0–1999–117 people (29.9%)—while 106 people (27.1%) earned between RMB 2000 and 3999. There were 252 people who often shopped through live streaming in the TikTok app (64.5%) and 139 people who often shopped through live streaming in the Taobao app (35.5%).

To avoid nonresponse bias, we performed a paired t-test on the demographic data of the first and last thirty people who answered the questionnaire. The results showed no significant difference; therefore, nonresponse was not a serious problem in this study.

Common method bias is a common issue in questionnaires, and two methods were used to measure it in this study. First, Harman’s single-factor analysis was conducted [80]. The results showed that the percentage of extracted single variables was 26.10% (less than 40%). The common method bias in PLS-SEM was measured according to FULL-VIF [79, 81], in which all VIF values were below 3.3. The results of both testing methods indicated that common method bias was not a serious problem.

### 4.4 Measurement model

To evaluate the measurement model, we evaluated composite reliability (CR), average variance extracted (AVE), discriminant validity, and outer loading. As shown in Table 2, the variables’ composite reliability was >0.7, and Cronbach’s alpha was >0.7, indicating that the internal consistency of the data in this study is satisfactory. The AVE value of >0.5 and outloadings of >0.7 indicate that the convergent validity of the data is also acceptable [77]. Discriminant validity was measured using Fornell and Larcker’s test and the heterotrait-monotrait ratio (HTMT) test. As shown in Table 3, the HTMT values were below the 0.85 threshold and the square root of each variable’s AVE was also greater than the correlation between variables [77]. Cross-loading is an additional criterion for measuring discriminant validity, and the results are presented in supporting information S3 Appendix. The results indicate that this study has good reliability, convergent validity, and discriminant validity.

**Table 2:** Reliability and validity of constructs.

Latent variable	Items	Loading	Mean (SD)	Cronbach’s a	CR	AVE
UVU	UVU1	0.894	2.519(0.892)	0.779	0.872	0.694
	UVU2	0.805				
	UVU3	0.798				
HVU	HVU1	0.724	2.579(0.923)	0.770	0.864	0.682
	HVU2	0.932				
	HVU3	0.809				
EEY	EEY1	0.916	2.700(0.956)	0.831	0.898	0.747
	EEY2	0.827				
	EEY3	0.847				
RCT	RCT1	0.922	3.422(0.949)	0.833	0.900	0.751
	RCT2	0.822				
	RCT2	0.853				
PII	PII1	0.891	3.680(0.689)	0.768	0.860	0.673
	PII2	0.775				
	PII2	0.791				
IOD	IOD1	0.914	3.153(0.919)	0.808	0.887	0.725
	IOD2	0.838				
	IOD3	0.797				
CRE	CRE1	0.912	2.623(0.741)	0.750	0.855	0.664
	CRE2	0.717				
	CRE2	0.805				

**Abbreviations:** UVU, utilitarian value uncertainty; HUV, hedonic value uncertainty; EEY, experiential efficacy; RCT, response cost; PII, stop purchase intention; IOD, information overload; CRE, consumer resilience.

**Table 3:** Discriminant validity.

Fornell-Larcker Criterion							
Items	UVU	HVU	EEY	RCT	PII	IOD	CRE
UVU	**0.833**						
HVU	-0.038	**0.826**					
EEY	0.361	-0.004	**0.864**				
RCT	-0.119	0.025	-0.116	**0.866**			
PII	0.323	-0.004	0.239	-0.393	**0.820**		
IOD	-0.388	-0.071	-0.165	0.374	-0.284	**0.851**	
CRE	-0.468	0.01	-0.212	0.512	-0.424	0.404	**0.815**
Heterotrait-Monotrait Ratio							
UVU							
HVU	0.058						
EEY	0.444	0.061					
RCT	0.147	0.042	0.136				
PII	0.39	0.085	0.296	0.449			
IOD	0.484	0.092	0.192	0.448	0.335		
CRE	0.586	0.043	0.242	0.607	0.485	0.502	

**Abbreviations:** UVU, utilitarian value uncertainty; HUV, hedonic value uncertainty; EEY, experiential efficacy; RCT, response cost; PII, stop purchase intention; IOD, information overload; CRE, consumer resilience.

### 4.5 Structural model

**Fig 2:**
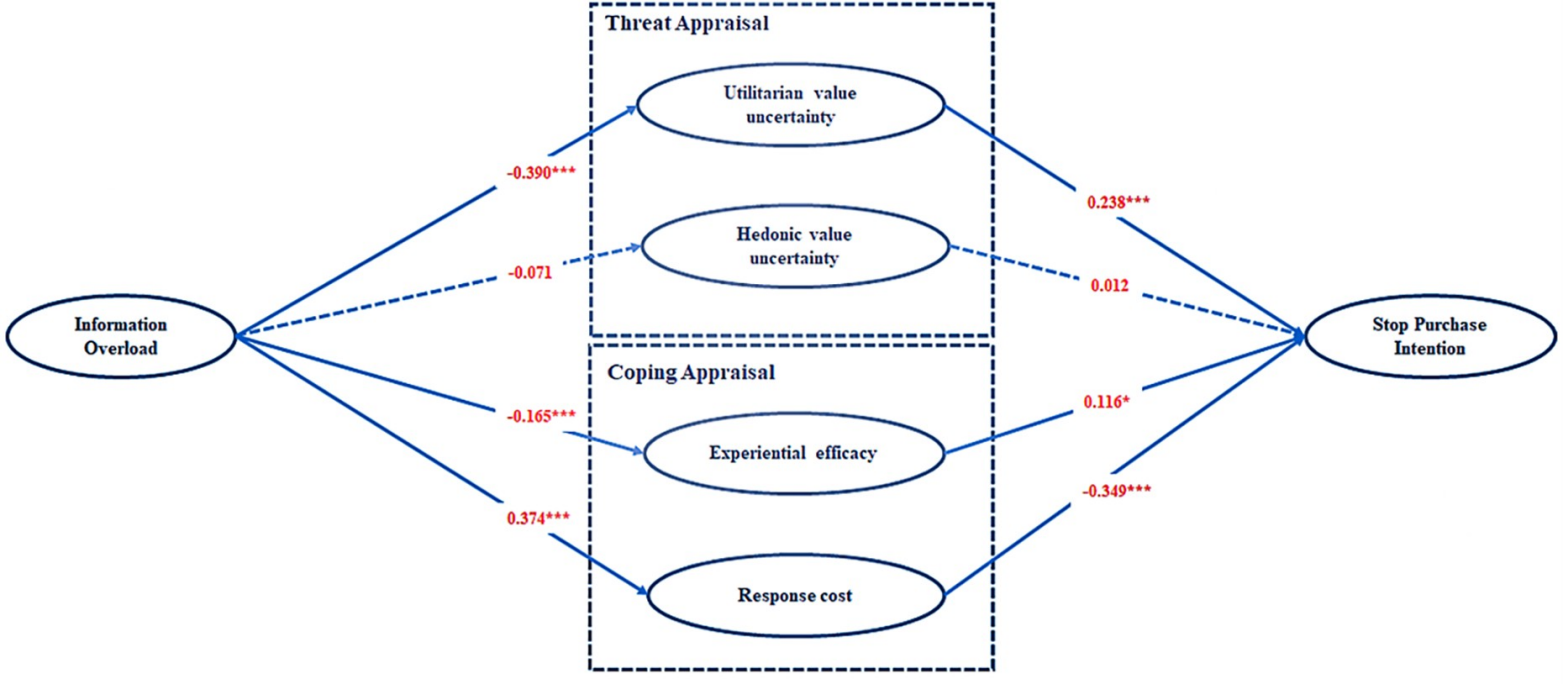
Structural model assessment results.

First, we checked for collinearity. The VIF values of the variables were all below 5; therefore, collinearity was not a major issue in this study. After ensuring that the reliability, validity, and collinearity of the model were not a problem, we analyzed the structural model to verify the hypothesis. The path coefficients and significance test results of the structural model are presented in Table 4 and Fig 2. Utilitarian value uncertainty (β = 0.238, p < 0.001) and experiential efficacy (β = 0.116, p < 0.05) have a significant positive effect on consumers’ intention to stop purchase; thus, H1a and H2a are supported. Response cost (β = -0.349, p < 0.001) has a significant negative effect on consumers’ intention to stop purchase, supporting H2b. Information overload has a significant negative effect on utilitarian value uncertainty (β = -0.390, p < 0.001) and experiential efficacy (β = -0.165, p < 0.01), supporting H3a and H4a. Information overload has a significant positive effect on response cost (β = 0.374, p < 0.001), supporting H4b. However, the effect of hedonic value uncertainty (β = 0.012, p>0.05) on consumers’ intention to discontinue a purchase is not significant; thus, H1b is not supported. The effect of information overload on hedonic value uncertainty (β = -0.071, p < 0.064) is not significant; therefore, H3b is also not supported. This study also measured the effect of control variables (subject’s age, gender, income, education level, and platform use) on stop purchase intention, and none of them were significantly related. In addition, there was no significant effect of different product categories on stop purchase intention.

**Table 4:** Assessment of the structural model.

Hypothesis	β	STDEV	T Statistics	P Values	Result
H1a: UVU -> PII	0.238	0.044	5.425	0.000	Support
H1b: HVU -> PII	0.012	0.058	0.202	0.840	Reject
H2a: EEY -> PII	0.116	0.048	2.433	0.015	Support
H2b: RCT -> PII	-0.349	0.04	8.639	0.000	Support
H3a: IOD -> UVU	-0.390	0.042	9.359	0.000	Support
H3b: IOD -> HVU	-0.071	0.064	1.103	0.270	Reject
H4a: IOD -> EEY	-0.165	0.050	3.282	0.001	Support
H4b: IOD -> RCT	0.374	0.045	8.216	0.000	Support
Gender	-0.028	0.047	0.595	0.552	-
Age	-0.027	0.044	0.613	0.540	-
Education	-0.027	0.040	0.679	0.497	-
Income	0.003	0.042	0.059	0.953	-
Platform	-0.013	0.044	0.306	0.760	-
Product	-0.050	0.045	1.105	0.269	-

**Abbreviations:** UVU, utilitarian value uncertainty; HUV, hedonic value uncertainty; EEY, experiential efficacy; RCT, response cost; PII, stop purchase intention; IOD, information overload; CRE, consumer resilience.

Finally, we tested the goodness of fit of the model. We used the standardized root mean square residual (SRMR) values to check the goodness of fit of the model in this study. The SRMR value was 0.063, which met the requirement of being less than the threshold value of 0.08. We conclude that the fit of this study is satisfactory [77].

### 4.6 Moderation effects

In this study, "consumer resilience" was proposed as a moderator and its moderating effect was tested. The test was performed in two parts. First, the significance of the moderating effect was measured. Following this, the strength of the moderating effect was measured by calculating F^2^, which was calculated as (R^2^interaction model–R^2^main effects model) / (1–R^2^main effects model). An F^2^ value between 0.02 and 0.15 indicates a small moderating effect, a value between 0.15 and 0.35 indicates a moderate moderating effect, and a value greater than 0.35 indicates a high moderating effect [82].

**Fig 3:**
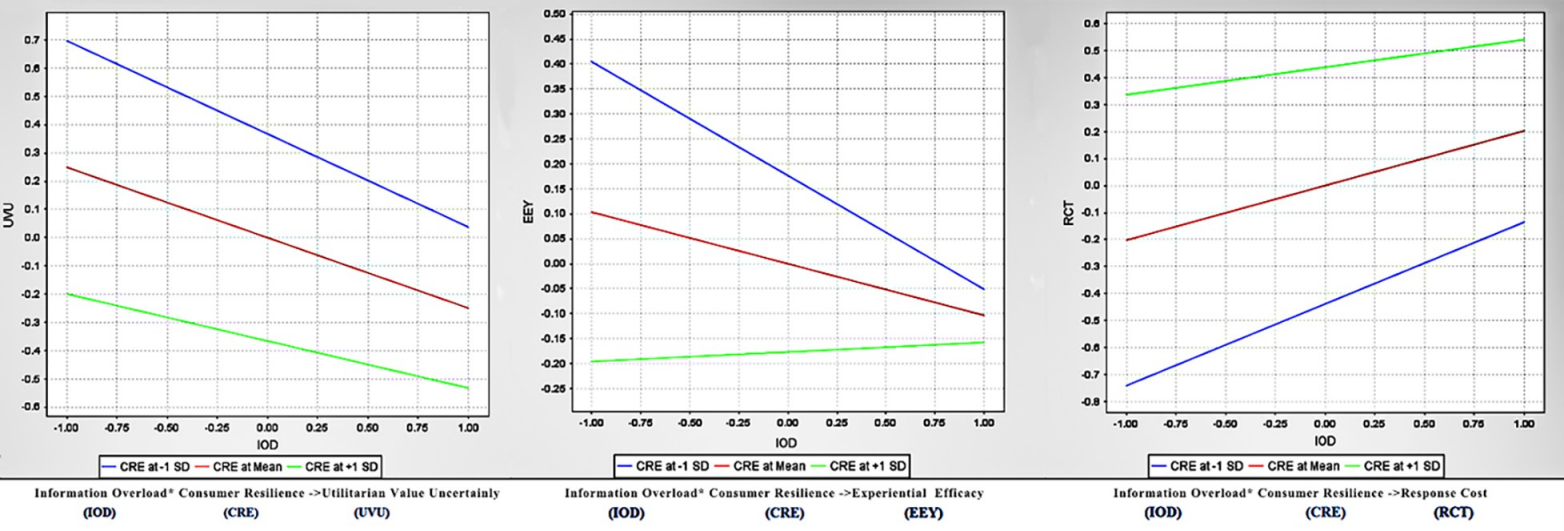
Simple slope analysis. **Abbreviations:** UVU, utilitarian value uncertainty; HUV, hedonic value uncertainty; EEY, experiential efficacy; RCT, response cost; IOD, information overload; CRE, consumer resilience.

The moderation effects are shown in Table 5 and Fig 3. Consumer resilience significantly moderates the negative effect of information overload on utilitarian value uncertainty (β = 0.082, p<0.05) with a small effect size (F^2^ = 0.143). Consumer resilience also significantly moderates the negative effect of information overload on experiential efficacy (β = 0.124, p<0.01), with a small effect size (F^2^ = 0.047). Consumer resilience significantly moderates the positive effect of information overload on response costs (β = -0.100, p<0.05), and the effect was medium (F^2^ = 0.203). There was no significant moderating effect of consumer resilience on information overload and hedonic uncertainty (β = -0.100, n.s).

**Table 5:** Moderation effect test.

Hypothesis	R^2^main effects model	R^2^interaction model	F^2^	β	T Statistics	Result
H5a: IOD*CRE ->UVU	0.152	0.273	0.143	0.082	2.123*	Support
H5b: IOD*CRE->HVU	0.005	0.013	0.008	0.020	0.328	Reject
H5c: IOD*CRE-> EEY	0.027	0.073	0.047	0.124	2.788**	Support
H5d: IOD*CRE ->RCT	0.140	0.315	0.203	-0.100	2.413*	Support

**Note:**

*p<0.05

**p<0.01

**Abbreviations:** UVU, utilitarian value uncertainty; HUV, hedonic value uncertainty; EEY, experiential efficacy; RCT, response cost; PII, stop purchase intention; IOD, information overload; CRE, consumer resilience.

## 5. Discussion and conclusion

### 5.1 Key findings

In this study, we have meticulously explored the intricate dynamics of consumer behavior within the sphere of live commerce, guided by the foundational principles of Protection Motivation Theory. Our investigation reveals that both threat and coping appraisals serve as essential mechanisms through which consumers navigate their purchasing decisions, particularly under the influence of the live streamers’ interactive strategies. Notably, we found that the uncertainty regarding product quality significantly influences consumers’ hesitation and propensity to abort potential purchases, highlighting the critical role of perceived product value in the digital shopping realm. Moreover, our findings illuminate the profound impact of past purchasing experiences on consumer behavior; positive experiences tend to bolster consumers’ confidence and likelihood to engage in repeat purchases, while negative experiences enhance their protective instincts, deterring future purchasing activities. This behavioral pattern is accentuated by the strategies employed by streamers, who often emphasize product value to stimulate spending, effectively mitigating information asymmetry and enhancing consumer purchase intentions. Interestingly, our research also uncovered some divergences from existing studies, particularly concerning the influence of hedonic value uncertainty and information overload, which did not significantly affect purchase decisions as previously theorized. This discrepancy may stem from the unique characteristics of the live commerce platforms in China, where the distinction between e-commerce and entertainment-focused platforms shapes consumer expectations and engagement levels. Our simulated live streaming marketing design, involving streamers and consumers without prior interaction, likely did not replicate the depth of emotional engagement typically observed on entertainment-centric platforms, thereby influencing the outcomes.

The following discussion will focus on several specific objectives of this study. The primary objective of this investigation was to elucidate the mechanisms by which consumers safeguard themselves from erroneous purchases within the realm of live commerce. Drawing upon Protection Motivation Theory, this study delineates how threat and coping appraisals act as pivotal determinants in fostering protection motivation among consumers [30]. Within the specific milieu of live commerce, our analysis substantiates that these appraisals can be discerned through more granular factors, which in turn, orchestrate a consumer protection mechanism, influencing their purchasing decisions. Our empirical findings illuminate that product uncertainty exerts a positive influence on consumers’ inclination to halt purchases. Such uncertainty, recognized as a utilitarian value in the digital shopping domain, amplifies consumers’ hesitancy regarding the congruence between product quality and their requirements, thereby diminishing their purchasing intent—a phenomenon that is accentuated in online shopping environments [6]. This apprehension, born out of uncertainty, propels consumers towards developing a self-protection intent. Furthermore, this study elucidates the role of coping appraisal, where the impact of experience effectiveness and response costs are meticulously examined. Aligning with Lin [56] observations, our findings suggest that consumers with positive past purchasing experiences are more predisposed to repeat purchases. Conversely, negative experiences galvanize a robust self-protection stance, potentially curtailing future purchasing behaviors. Additionally, the study delves into response costs, a crucial element in the consumer’s coping appraisal, emphasizing how streamers in live commerce frequently accentuate the added value of products to stimulate consumer spending [13]. In concordance with Xu, Cui and Lyu [26], our study corroborates the notion that real-time interaction in live commerce can mitigate information asymmetry, thereby enhancing consumers’ purchase intentions by alleviating product uncertainties. Concurrently, our insights on response costs find a parallel in Chen and Yang [70] discourse on the significance of network structural embeddedness in shaping consumer purchase intentions in cross-border e-commerce, offering a complementary viewpoint on consumer decision-making online. Moreover, our analysis on the efficacy of past experiences finds resonance with Helin, Donglu, Shaoying, Decheng and Bei [27] investigation into the impact of online comments and merchant responses on tourism product sales, underscoring the interplay between various facets of online commerce. The study by Zhang, Yang and Bei [18] further enriches our discussion, presenting a nuanced perspective on how psychological ownership and social capital influence consumer behavior in virtual communities, thereby offering strategic insights to mitigate incorrect purchasing in live commerce. This comprehensive discussion, grounded in empirical evidence and theoretical frameworks, not only amplifies our understanding of consumer self-protection mechanisms in live commerce but also establishes a confluence with extant research, thereby contributing to the broader discourse on consumer behavior in online shopping environments.

The Second objective of the present study was to investigate the potential of information overload, propagated by live streamers in e-commerce environments, to circumvent the mechanisms consumers employ to protect themselves from erroneous purchases. The analysis conducted confirms that information overload does, indeed, compromise these self-protection mechanisms by attenuating threat appraisal, thereby reducing consumer uncertainty about the practical value of products. This finding aligns with the research conducted by Xu, Cui and Lyu [26] which suggested that the direct interaction between streamers and consumers during live sessions may decrease information asymmetry and increase the intention to purchase. However, the authors also highlighted the critical influence of streamer professionalism and the parasocial relationship with the viewer on this dynamic. Additionally, our findings indicate that information overload can weaken coping appraisals within consumer self-protection frameworks, evidenced by decreased effectiveness of experience and increased consumer response costs. This observation is consistent with the study by Wei, Hai, Zhu and Lyu [25], which examined the effect of consumers’ deferral of choices on their preferences in intertemporal decision-making, emphasizing the vital role of perceived information integrity in influencing consumer preferences. Complementing our insights, the application of the stimulus-organism-response model by Zhang, Qi and Lyu [9] illustrates how knowledge sharing within virtual communities can shape consumer-brand relationships, thereby underscoring the significance of information quality and community interaction in influencing consumer perceptions and decision-making processes. Furthermore, our exploration into the direct impacts of information overload is enriched by the findings of Zhang, Yang and Bei [18], who investigated the roles of social capital and psychological ownership in virtual communities on knowledge sharing among consumers. Their study suggests an indirect influence on consumer decision-making and a potential buffering effect against information overload. In essence, these studies collectively illuminate the intricate dynamics between information presentation, consumer perception, and decision-making in the realm of digital commerce. They underscore the necessity of a balanced information delivery approach and the cultivation of positive consumer relationships and vibrant community interactions to counteract the detrimental effects of information overload. Such strategies not only aid in consumer decision-making but also enhance the overall efficacy of live commerce platforms. Notably, streamers’ creation of a false sense of urgency and the subsequent consumer difficulty in accurately assessing product value, as highlighted by Cao, Liu, Shang and Zhou [59], Soroya, Farooq, Mahmood, Isoaho and Zara [66], Sun, Shao, Li, Guo and Nie [2], Wongkitrungrueng and Assarut [7], and Zhang, Sun, Qin and Wang [13], accentuates the critical need for mitigating these effects to foster informed consumer choices and enhance the integrity of live commerce environments.

The third objective of our research was to delve into how consumer resilience influences the reactivation of self-protection mechanisms against unsuitable purchases during instances of information overload from streamers in live commerce environments. Our results substantiate the critical function of resilience in alleviating information-induced stress, echoing [28] observations that, within the live shopping sphere, the deluge of information tends to erode consumer resistance to temptation, yet resilience can counterbalance this effect by diminishing the impact of information overload. Additionally, our investigation validates that resilience mitigates the negative ramifications of information overload on the uncertainty associated with a product’s practical value and on the effectiveness of experiences, while concurrently having a beneficial impact on response costs. This aligns with the findings of Helin, Donglu, Shaoying, Decheng and Bei [27], who unravel the intricate interplay between online feedback, merchant reactions, and the sales metrics of tourism offerings, underlining the capacity of consumer engagement strategies to shape perceptions and influence decision-making. Such evidence intimates that resilience, similar to proactive consumer engagement, may act as a protective mechanism within the information processing continuum, augmenting the consumer’s proficiency in selectively assimilating and evaluating information. Furthermore, the analysis conducted by Zhang, Qi and Lyu [9] utilizing the stimulus–organism–response paradigm within virtual communities accentuates the manner in which external stimuli, such as information overload, can affect consumer-brand relationships. Their insights into the importance of the quality of knowledge exchange in molding consumer perceptions highlight the utility of resilience in aiding consumers to traverse information-dense environments more adeptly, thus fostering more robust consumer-brand connections despite the prevalence of information overload. Moreover, the research by Xu, Cui and Lyu [26] concerning the interplay between a streamer’s professionalism and the parasocial relationship with viewers in live commerce provides an illustrative backdrop wherein consumer resilience may be particularly advantageous. In scenarios where the professional conduct of streamers and relational dynamics are pivotal, resilience emerges as a key trait enabling consumers to critically assess information, thereby bolstering their decision-making capabilities amidst persuasive tactics utilized by streamers. This discourse underlines the indispensability of resilience in the contemporary consumer’s toolkit, offering a shield against the barrage of information inherent in the digital commerce landscape.

In this study, we encountered findings that diverge from existing research, particularly in the domain of consumer behavior in live commerce settings. Firstly, the anticipated influence of hedonic value uncertainty on disrupting purchase decisions did not align with the established consumer protection mechanisms against incorrect purchases identified in prior studies. Secondly, the anticipated adverse impact of information overload on hedonic value uncertainty failed to materialize in our analysis. This discrepancy invites a nuanced interpretation, possibly tied to the distinct nature of live commerce platforms in China, as identified by Cai, Wohn, Mittal and Sureshbabu [50]. The landscape of live commerce in China bifurcates into two primary categories: platforms that are extensions of traditional e-commerce services and those that evolve from live streaming entertainment platforms. This distinction is not trivial, as it underpins the varying consumer motivations across these platforms. On e-commerce-centric platforms, the consumer’s focus is predominantly on the utilitarian aspects of the product. Conversely, on platforms with roots in live streaming entertainment, the hedonic value derived from interactions with streamers tends to take precedence. The simulated live streaming marketing design of our study, which involved streamers and consumers who were unfamiliar with each other prior to the experiment, might not have effectively replicated the depth of emotional engagement typically observed on live streaming entertainment platforms. Despite efforts to induce hedonic value through designed information stimuli, the absence of pre-existing emotional connections likely attenuated the potential for forming strong attachment and trust bonds within the limited timeframe of the simulated live streaming marketing. Given that the simulated live streaming marketing setup mirrored the context of an e-commerce platform incorporating live commerce features, participant focus was likely skewed towards product utility rather than hedonic value. Consequently, the anticipated influence of hedonic value uncertainty on purchase interruptions did not manifest significantly, suggesting that the context and nature of consumer-streamer interactions play a critical role in shaping purchase behaviors in live commerce environments.

### 5.2 Theoretical contributions

First, the model proposed in this study confirms the existence of an offensive and defensive game between streamers and consumers in commercial marketing, which provides a new theoretical perspective regarding the operation of live commerce and enriches the marketing literature. (1) Consumers measure the level of potential harm from a product purchase during live stream by assessing the uncertainty in utilitarian value and then decide whether to stop purchase by combining experience effectiveness and response cost, to protect themselves from being harmed by a wrong purchase. (2) The streamer can effectively break through the consumer’s self-protection mechanism by using an information overload strategy. However, consumer resilience can also mitigate the impact of this strategy.

Second, PMT was applied to live commerce to analyze the protection psychology of consumers, thereby expanding the scope of application of PMT. The results showed that (1) consumers can measure the degree of harm from wrong purchases by assessing a product’s utilitarian value uncertainty in live commerce, which enriches the dimension of threat appraisal in PMT; (2) consumers assess their ability to adopt protective behaviors by weighing the pros and cons of adopting protective behaviors based on their experience and existing abilities, which enriches the dimension of coping appraisal in PMT.

Third, the important role of information overload in the field of marketing was confirmed by introducing information overload into PMT, which extends the scope of application of information overload theory and enriches the literature in the field of marketing. (1) The effect of information overload on consumer uncertainty about product value was confirmed, enriching the antecedents of threat appraisal in PMT. (2) The effect of information overload on experiential efficacy and response cost was confirmed, which enriches the antecedents of coping appraisal in PMT.

Finally, this study makes some contributions to resolving the controversy over the relationship between information overload and consumers’ purchase intentions. 1) Previous studies have pointed out that information overload can both positively influence consumers’ purchase intentions[19, 20], and negatively affect them [19, 21], From the results of this study, the reason for this controversy can be explained as follows: consumers have a psychological defense mechanism, when the information overload marketing from the seller is sufficient to break the consumer’s psychological defense mechanism, it actively pushes the consumer to purchase; if the information overload is not sufficient to break the consumer’s defense mechanism, the consumer will not purchase. 2) Previous studies have also suggested an inverted U-shaped relationship between information overload and consumers’ purchase intentions[22, 62], The results of this study can also explain such results: When information overload is low, users can make high-quality decisions, purchase intentions are less influenced by marketing, and purchase decisions are rational; When the degree of information overload reaches a certain level, the quality of users’ decisions gradually decreases, and they gradually become more influenced by sellers’ marketing and are easily prone to make irrational purchases. However, because of the existence of consumer resilience, consumers will gradually get used to the information overload, even if the information overload given by sellers becomes higher and higher, the impact on consumers is more and more limited, and consumers’ purchase decisions tend to be rational again. In addition, this results also confirm that consumer resilience can weaken the impact of merchant marketing. This provides a new perspective in the study of resilience in the field of marketing and enriches the PMT.

### 5.3 Practical contributions

The findings of the research underscore the nuanced interplay between streamer strategies and consumer responses within the live commerce context, highlighting the need for a holistic and ethical approach to enhance both the effectiveness and the ethical standards of live commerce. Streamers play a crucial role in shaping the consumer experience, and their actions can either empower consumers or lead them into decision-making traps spurred by information overload.

To navigate this delicate balance, streamers require advanced training that goes beyond mere communication skills and script usage. They need to be imbued with ethical marketing practices, ensuring they present information in a way that is clear, engaging, and not overwhelming. This approach helps in creating an environment where consumers are informed and involved, rather than being led into an information prison where they are more susceptible to making impulsive or uninformed decisions.

On the other side, empowering consumers is equally vital. The research findings suggest that consumers equipped with the right knowledge and tools can critically assess the information presented to them during live commerce sessions. Awareness campaigns and educational initiatives are crucial in helping consumers recognize signs of information overload and guiding them to use external platforms for additional verification of product details. Such informed consumers are more resilient to aggressive marketing tactics and can make autonomous purchasing decisions.

Resilience among consumers emerges as a key theme in the study, indicating that when consumers have resources to enhance their knowledge and decision-making capabilities, they are better positioned to withstand marketing pressures. This resilience is further supported when live-commerce platforms and streamers commit to ethical marketing practices, prioritizing consumer well-being and ensuring transparency and honesty in their communications.

Continuous monitoring and gathering consumer feedback are essential to ensure that live commerce evolves in a direction that aligns with consumer preferences and tolerances for information. By integrating these strategies, live commerce can strike an optimal balance between engaging marketing and consumer well-being, creating an environment conducive to informed choices and respected consumer autonomy.

Thus, the research findings advocate for a comprehensive approach that incorporates streamer training, consumer empowerment, resilience building, ethical marketing, and continuous feedback. This approach not only enhances the effectiveness of live commerce but also upholds its ethical standards, ensuring a sustainable and consumer-friendly live commerce ecosystem.

### 5.4 Limitations and future directions

While this study provides valuable insights into consumer behavior and streamer interactions in live commerce, it also presents several limitations that future research should address. Firstly, the simulated live streaming marketing design, particularly the simulation environment for live shopping, lacked emotional bonds between streamers and consumers. The participants were not familiar with the streamers, who were not prominent Internet celebrities. This setup overlooked the critical ’fan effect’ in live commerce, which could influence consumption motivations tied to hedonic values. Future studies should consider the simulated live streaming marketing involving well-known personalities to better capture this dynamic.

Additionally, the study did not account for interactions among consumers, which can be a significant factor in live commerce environments. Consumer-to-consumer interactions can sometimes mitigate or exacerbate the effects of information overload, suggesting that future research should explore this aspect more thoroughly. The study also focused solely on the streamer-to-consumer relationship, neglecting other forms of overload such as system, communication, and social overload, which warrant further investigation.

In terms of consumer protection mechanisms, this study primarily drew from protection motivation theory to identify key factors. However, there might be other relevant theories and factors that could provide a more comprehensive understanding of consumer behavior in live commerce settings. Future research could explore additional theoretical frameworks to uncover other potential mechanisms of consumer protection. Furthermore, the study’s geographic and demographic scope was limited to Jiangsu Province, China, with a relatively small sample size that may not fully represent the broader population. This limitation raises concerns about the generalizability of the findings to other regions or demographics. Future research should aim to include a more diverse and larger sample to enhance the external validity of the findings.

## Supporting information

S1 Appendix Examples of customer communication script. (DOCX)

S2 Appendix Measurement items. (DOCX)

S3 Appendix Discriminant validity- cross loading. (DOCX)

S1 Data (CSV)
