# Supplementary material for: Offense and defense between streamers and customers in live commerce marketing: Protection motivation and information overload
Source: PLoS One. 2024 Sep 6;19(9):e0305585. doi: 10.1371/journal.pone.0305585 (PMC11379157; doi:10.1371/journal.pone.0305585)
Supplement: S1 Appendix — (DOCX) [file pone.0305585.s001.docx]

## Appendix A. Examples of customer communication script for experiment

| Stage | Main tasks | Speech speed and frequency | Examples of customer communication script |
| --- | --- | --- | --- |
| Interactive Stage  (Channel A) | 1. Greeting consumers  2.Warmly introduce the content of the upcoming live broadcast  3.Forecast various promotions to retain consumers | 1. Normal speech speed  2. Loud statements 1-2 times  3. Every new entry of 10 people will be done again | "Dear family members, we start the lottery at 8:00 be ready, speak actively and don't leave." "For those of you who want this item, type 1 in the comment section" |
| Product introduction stage  (Channel A)) | 1. Show product features, sales, efficacy.  2. Emphasize that the purchase of products can enhance the relationship with the streamer | 1. Normal speech speed  2. Speak aloud 1-2 times  3. If there is time to spare, circulate | Utilitarian value content：  1. "Wearing the clothes in winter, super warm"  2. "This dress is especially suitable for snow days, super good-looking"  3. "The clothes are windproof and waterproof fabric, both style quality and practicality are very good"  Hedonic value content：  "Buy this dress, you can enter the streamer's guardian group, not only after-sales, you can also chat and interact with the streamer " |
| Push stage  (Information overload marketing stage)  (Channel B) | 1. Repeat the content of the product introduction stage in different forms.  2. Repeatedly create a rush atmosphere.  3. Give the consumer the instruction to place an order quickly.  4. Clearly and repeatedly communicate 3-4 content to consumers in as short a time as possible | 1. Clear and fast speech speed  2. Enthusiastic and loud.  3. Repeat as many times as possible in a short time. | "Limited number of offers, will be sold out soon, hurry up and place your order"  "Limited number of free gifts today, first come, first served"  "We're the only ones with this price."  "Don't think twice, this product is perfect for your skin."  "Buy 2 bottles today and get 70 off, that's only 45 a bottle!".  "Buy this dress, you can also get the streamer's contact information and make friends with the streamer"  "Help the streamer, the streamer will sell 10 more pieces to complete the task, the streamer can have a good year"  "Every time you buy a product, you are contributing to the schooling of poor children in the mountains." |
