# Supplementary material for: Offense and defense between streamers and customers in live commerce marketing: Protection motivation and information overload
Source: PLoS One. 2024 Sep 6;19(9):e0305585. doi: 10.1371/journal.pone.0305585 (PMC11379157; doi:10.1371/journal.pone.0305585)
Supplement: S2 Appendix — (DOCX) [file pone.0305585.s002.docx]

## Appendix B. Measurement items

| **Factors** | **Serial Num.** | **Item** | **Reference** |
| --- | --- | --- | --- |
| Utilitarian Value Uncertain | UVU1 | I am concerned that these products will not work as advertised by the streamers in real life. | Lu & Chen (2021)  Park & Lin (2020) |
|  | UVU2 | I'm not sure if these products will work on me as well as I expect them to. |  |
|  | UVU3 | The streamer's recommendation did not allow me to determine the true characteristics of the product. |  |
| Hedonic Value Uncertain | HVU1 | I'm not sure buying a product recommended by the streamer would make me happy | Lu & Chen (2021)  Park & Lin (2020) |
|  | HVU2 | I'm not sure that enhancing my relationship with the host by purchasing the product will make me happy |  |
|  | HVU3 | I'm not sure the streamer recommended products are interesting |  |
| Experience Efficacy | EEY1 | Based on shopping experience, I can interrupt the purchase | Farooq et al. (2021)  Tsai et al. (2016) |
|  | EEY2 | Based on shopping experience, it is easy to interrupt a purchase |  |
|  | EEY3 | Based on shopping experience, I interrupt the purchase is necessary |  |
| Response Cost | RCT1 | I believe that the cost of taking measures to interrupt the purchase is higher than the benefit of continuing the purchase. | Farooq et al. (2021) |
|  | RCT2 | I don't encourage myself to interrupt my purchase, it makes me lose the discount. |  |
|  | RCT3 | I'd look silly if I didn't keep buying. |  |
| Purchase Interruption Intention | PII1 | To prevent wrong purchase, I will stop buying the products recommended by the streamer | Park & Lin (2020) |
|  | PII2 | The product recommended by the streamer may not be worth my purchase, I will stop buying |  |
|  | PII3 | I would not recommend such a product to friends. |  |
| Information Overload | IOD1 | I was distracted by the amount of product information I received from the streamer | Farooq et al. (2021) |
|  | IOD2 | want to deal with the large amount of information coming from the streamer |  |
|  | IOD3 | I can't synthesize the information given by the streamer. |  |
| Consumer Resilience | CRE1 | I am optimistic about the pressure I am under | Bermes (2021) |
|  | CRE2 | In the face of bad things, it is easier for me to return to normal |  |
|  | CRE3 | It doesn't take me long to get over the frustrations in my life |  |
