# Supplementary material for: Offense and defense between streamers and customers in live commerce marketing: Protection motivation and information overload
Source: PLoS One. 2024 Sep 6;19(9):e0305585. doi: 10.1371/journal.pone.0305585 (PMC11379157; doi:10.1371/journal.pone.0305585)
Supplement: S3 Appendix — (DOCX) [file pone.0305585.s003.docx]

## Appendix C. Discriminant Validity- Cross Loading

|  | CRE | EEY | HVU | IOD | PII | RCT | UVU |
| --- | --- | --- | --- | --- | --- | --- | --- |
| CRE1 | **0.912** | -0.261 | 0 | 0.41 | -0.475 | 0.58 | -0.492 |
| CRE2 | **0.717** | -0.091 | -0.027 | 0.239 | -0.172 | 0.262 | -0.271 |
| CRE3 | **0.805** | -0.119 | 0.049 | 0.305 | -0.317 | 0.33 | -0.333 |
| EEY1 | -0.213 | **0.916** | 0.039 | -0.195 | 0.214 | -0.106 | 0.366 |
| EEY2 | -0.17 | **0.827** | -0.011 | -0.092 | 0.188 | -0.059 | 0.276 |
| EEY3 | -0.162 | **0.847** | -0.046 | -0.126 | 0.217 | -0.13 | 0.283 |
| HVU1 | 0.004 | -0.054 | **0.724** | -0.034 | -0.019 | 0.039 | -0.045 |
| HVU2 | 0.009 | 0.028 | **0.932** | -0.076 | 0.011 | 0.012 | -0.032 |
| HVU3 | 0.011 | -0.013 | **0.809** | -0.053 | -0.015 | 0.022 | -0.023 |
| IOD1 | 0.339 | -0.184 | -0.064 | **0.914** | -0.237 | 0.36 | -0.334 |
| IOD2 | 0.354 | -0.135 | -0.091 | **0.838** | -0.288 | 0.29 | -0.35 |
| IOD3 | 0.341 | -0.096 | -0.023 | **0.797** | -0.198 | 0.301 | -0.306 |
| PII1 | -0.438 | 0.214 | -0.035 | -0.305 | **0.891** | -0.434 | 0.346 |
| PII2 | -0.228 | 0.174 | -0.04 | -0.161 | **0.775** | -0.186 | 0.178 |
| PII3 | -0.317 | 0.196 | 0.071 | -0.19 | **0.791** | -0.271 | 0.221 |
| RCT1 | 0.434 | -0.104 | 0.029 | 0.391 | -0.36 | **0.922** | -0.102 |
| RCT2 | 0.419 | -0.084 | 0.024 | 0.257 | -0.324 | **0.822** | -0.102 |
| RCT3 | 0.481 | -0.113 | 0.012 | 0.309 | -0.335 | **0.853** | -0.106 |
| UVU1 | -0.414 | 0.324 | -0.038 | -0.403 | 0.277 | -0.115 | **0.894** |
| UVU2 | -0.363 | 0.275 | -0.031 | -0.269 | 0.289 | -0.072 | **0.805** |
| UVU3 | -0.394 | 0.301 | -0.025 | -0.286 | 0.244 | -0.109 | **0.798** |

**Abbreviations:** UVU, utilitarian value uncertainty; HUV, hedonic value uncertainty; EEY, experiential efficacy; RCT, response cost; PII, stop purchase intention; IOD, information overload; CRE, consumer resilience.
